# High specific selectivity and Membrane-Active Mechanism of the synthetic centrosymmetric α-helical peptides with Gly-Gly pairs

**DOI:** 10.1038/srep15963

**Published:** 2015-11-04

**Authors:** Jiajun Wang, Shuli Chou, Lin Xu, Xin Zhu, Na Dong, Anshan Shan, Zhihui Chen

**Affiliations:** 1 Institute of Animal Nutrition, Northeast Agricultural University, Harbin 150030, P.R. China

## Abstract

We used a template-assisted approach to develop synthetic antimicrobial peptides, which differ from naturally occurring antimicrobial peptides that can compromise host natural defenses. Previous researches have demonstrated that symmetrical distribution patterns of amino acids contribute to the antimicrobial activity of natural peptides. However, there is little research describing such design ideas for synthetic α-helical peptides. Therefore, here, we established a centrosymmetric α-helical sequence template (y + hhh + y)_n_ (h, hydrophobic amino acid; +, cationic amino acid; y, Gly or hydrophobic amino acid), which contributed to amphipathicity, and a series of centrosymmetric peptides was designed with pairs of small amino acids (Ala and Gly), which were utilized to modulate the biological activity. The centrosymmetric peptides with 3 repeat units exhibited strong antimicrobial activity; in particular, the Gly-rich centrosymmetric peptide GG3 showed stronger selectivity for gram-negative bacteria without hemolysis. Furthermore, beyond our expectation, fluorescence spectroscopy and electron microscopy analyses indicated that the GG3, which possessed poor α-helix conformation, dramatically exhibited marked membrane destruction via inducing bacterial membrane permeabilization, pore formation and disruption, even bound DNA to further exert antimicrobial activity. Collectively, the Gly-rich centrosymmetric peptide GG3 was an ideal candidate for commercialization as a clinical therapeutic to treat gram-negative bacterial infections.

The widespread increase in bacterial resistance to many conventional antibiotics has become a worldwide problem. Despite the abuse of antibiotics has been strictly restricted, the continuous increasing prevalence of multidrug-resistant bacteria still makes people highly nervous. Furthermore, the multidrug-resistant bacteria infections associated to medical implantation material are also threatening the human health, but for example, urinary catheter coatings are unable to provide sufficient protection against multidrug-resistant bacteria^1^. Therefore, the development of alternative antimicrobial coatings materials against multidrug-resistant bacteria is extremely urgent. One potential source of novel antibiotics is Gene-encoded antimicrobial peptides (AMPs), evolutionarily ancient weapons against invading microbes, which are the first barrier of the innate immune system in most multicellular organisms^2^. AMPs have been isolated and characterized from practically all living organisms, ranging from prokaryotes to humans^3^. Currently, the known primary structures of AMPs vary widely, and more than 2400 sequences have been documented in the Antimicrobial Peptide Database (APD) (http://aps.unmc.edu/AP/main.php). The most abundant type of peptide is the α-helical AMP, with over 340 being reported (see the APD), and they appear to represent particularly successful structural arrangements in innate defense^4^. They have broad-spectrum antibiotic activity, kill bacteria by interacting with the bacterial cell membranes and differ from antibiotics that target specific molecular receptors of pathogens^5^. Consequently, it will be difficult for bacteria to evolve resistance to α-helical AMPs unless bacteria alter their cell membrane composition^6^. Based on the above mechanism of action, α-helical AMPs are an ideal candidate for replacing conventional antibiotics and have potential as antimicrobial coating materials for controlling implant-related infections, which aid in the development of new bioengineering and biomedical materials^7^.

Despite α-helical AMPs bring the hope and opportunity to overcome the drug-resistance bacteria, some shortcomings, such as systemic toxicity, ease of degradability, and high cost, still impede the further development of natural peptides as therapeutic drugs and peptide-based biomaterials. Various methods have been employed to modify and/or optimize the natural α-helical AMPs, which focus on improving antimicrobial activity, while reducing the undesirable cytotoxicity towards mammalian cells. But these methods are complicated, random and lack systematic design principles^8^. Moreover, some reports showed that widespread clinical use of AMPs with sequences that are too close to those of human AMPs would inevitably compromise own natural defenses^9^. In this view, synthetic AMPs are a viable alternative^10^.

The template-assisted approach is a promising method to guide synthetic AMPs design, which maintains evolutionarily conserved sequence patterns and reduces the number of the candidate AMPs required to obtain useful results. The first typical sequence templates were proposed by Tossi *et al*.^11^, which were obtained by comparing and analyzing the sequences of over 150 naturally occurring α-helical peptides^12^. By further comparatively analyzing the typical sequence templates, we found an interesting phenomenon in that the sequences of amino acids were nearly a centrosymmetric distributed sequence (Supplementary Fig. S1A and S1B). For these reasons, we prudently hypothesized that the centrosymmetric sequence might be a relic of evolutionary divergence that contributed to the antimicrobial activity. Some literatures have demonstrated part of our conjecture that the symmetrical distribution of amino acids is a promising strategy for optimizing the natural β-turn peptides with strong antimicrobial activity^7^,^13^,^14^. While there is little research describing such design ideas for α-helical peptides, whether derivative from natural peptides or synthetic peptides. Most of all, they do not establish a systematical and universally applicable centrosymmetric sequence template to guide the AMPs design, whether α-helix or β-turn.

Small amino acids (Ala and Gly) are always ignored or replaced in the modification and/or optimization process of the natural peptides^15^,^16^. However, some new ideas were put forward that the rational use of small amino acid substitutions can improve the biological properties of AMPs; in particular, selectivity (reduced cytotoxicity) is improved^17^,^18^. And the motifs of two small residues are present with high frequency in the transmembrane helical domains^19^. Therefore, In this study, we established a primary centrosymmetric α-helical sequence template based on the α-helical protein-folding principles to conserve amphipathicity, which is a necessary property of peptides interacting with target membranes^20^. The primary sequence of the centrosymmetric α-helical template is the (y + hhh + y)_n_ amino acid sequence (h, hydrophobic amino acid; +, cationic amino acid; y, Gly or hydrophobic amino acid) and a series of centrosymmetric peptides was designed with the pairs of small amino acids (Ala and Gly). Furthermore, the corresponding peptides with scrambled centrosymmetric sequences were also designed. All the peptides were first characterized by circular dichroism (CD) for their secondary conformation in phosphate buffer solution (PBS) and in membrane-mimicking environments (sodium dodecyl sulfate (SDS) and trifluoroethyl alcohol (TFE)). Then, the hemolytic properties, cytotoxicity towards mouse macrophage RAW264.7 and antimicrobial activity of peptides were measured *in vitro*; the salt sensitivity of the peptides was determined against model microbes, such as *Escherichia coli (E. coli)* ATCC 25922 and *Staphylococcus aureus (S. aureus)* ATCC29213. The capability of the peptides to synergize with antibiotics was evaluated using the checkerboard titration method. Flow cytometry, scanning electron microscopy (SEM) and transmission electron microscopy (TEM) allowed direct observation of the changes in cell morphology and the integrity of cell membranes after peptide treatment. Assays examining liposome leakage, outer membrane permeabilization and membrane depolarization were employed to investigate the membrane destruction mechanisms of the peptides, and DNA-binding assays were employed to investigate the cell death as well as the possible intracellular targets of the peptides.

## Materials and Methods

### Materials

The peptides designed in this study as well as the fluorescent labeled peptides were synthesized by GL Biochem Corporation (Shanghai, China), and we further confirmed the fidelity via matrix-assisted laser desorption/ionization time-of-flight mass spectroscopy (MALDI-TOF MS; Linear Scientific Inc., USA), using α-cyano-4-hydroxycinnamic acid as the matrix. The purity of the peptides (95%) was assessed by reverse-phase high-performance liquid chromatography (HPLC), and the peptides were further subjected to electrospray MS to confirm their molecular weight. The peptides were amidated at the C-terminus.

*E. coli* ATCC 25922, *Salmonella typhimurium (S. typhimurium)* ATCC14028, *S. aureus* ATCC29213, *Staphylococcus epidermidis (S. epidermidis)* ATCC12228, and *Methicillin-resistant S. aureus* ATCC 43300 were obtained from *the* College of Veterinary Medicine, Northeast Agricultural University (Harbin, China), and *E. coli* UB1005 was kindly provided from the State Key Laboratory of Microbial Technology (Shandong University, China). The murine macrophage cell line RAW264.7 was purchased from the cell bank of the Chinese Academy of Sciences, SIBS (Shanghai, China). Human red blood cells (hRBCs) were obtained from the Northeast Agricultural University Hospital.

Phosphate-buffered saline (PBS; Kermel, China), sodium dodecyl sulfate (SDS; Sigma-Aldrich, China) and trifluoroethyl alcohol (TFE; Amresco, USA) were used in CD measurements. Ciprofloxacin (Sigma-Aldrich, China), ceftazidime (Sigma-Aldrich, China), streptomycin (Sigma-Aldrich, China) and chloramphenicol (Kayou, China) were used in synergy assays. 1-palmitoyl-2-oleoyl-*sn*-glycero-3-phosphocholine (POPC, Avestin, Inc., Canada) and 1-palmitoyl-2-oleoyl-*sn*-glycero-3-[phospho-rac-(1-glycerol)] (sodium salt) (POPG, Avestin, Inc., Canada) were used in liposome leakage assay. Triton X-100 (Sigma-Aldrich, China), *N*-phenyl-1-napthylamine (NPN; Sigma-Aldrich, China) and 3, 3'-dipropylthiadicarbocyanine iodide (diSC_3_-5; Sigma-Aldrich, China) were used in membrane permeability assays. Mueller Hinton broth (MHB) powder and tryptic soy broth powder were purchased from AoboX (China) and used according to the manufacturer’s instructions. Dulbecco's modified eagle medium (DMEM) and fetal bovine serum (FBS) were purchased from Gibco (China). Colorimetric 3-(4,5-dimethylthiazol-2-yl)-2,5-diphenyltetrazolium bromide (MTT) dye, dimethyl sulfoxide (DMSO), 4-(2-hydroxyethyl)piperazine-1-ethanesulfonic acid (HEPES), ethanol (analytical grade, 99%), acetone (analytical grade, 99%), tert-butanol (analytical grade, 99%), glutaraldehyde (synthetic grade, 50% in H_2_O), ethylene diamine tetraacetic acid (EDTA) and propidium iodide (PI) were purchased from Sigma-Aldrich (China).

### Peptide sequence analysis

The primary sequence analysis of the centrosymmetric peptides were performed online using EMBOSS Pepinfo (http://www.ebi.ac.uk/Tools/seqstats/emboss_pepinfo/). The secondary structure type for each residue was predicted online using Jpred 3 (http://www.compbio.dundee.ac.uk/www-jpred/index.html). The helical wheel projection and relative hydrophobic moment were performed online using the Heliquest analysis (http://heliquest.ipmc.cnrs.fr/cgi-bin/ComputParamsV2.py).

### CD measurements

To study the secondary structures in different membrane environments, the peptide solutions with a final concentration of 150 μM were prepared in 10 mM PBS (pH 7.4,mimicking an aqueous environment), 50% TFE (mimicking the hydrophobic environment of the microbial membrane), and 30 mM SDS micelles (a condition comparable to the negatively charged prokaryotic membrane)^7^. CD spectra were measured at 25 °C on a J-820 spectropolarimeter (Jasco, Tokyo, Japan), using a quartz cell with1.0 mm path length. The spectra were recorded from 190 to 250 nm at a scanning speed of 10 nm min^−1^ and averaged >3 runs for each sample. The acquired CD spectra were then converted to the mean residue ellipticity by using the following equation:


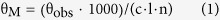


where θ_M_ is residue ellipticity (deg cm^2^ dmol^−1^), θ_obs_ is the observed ellipticity corrected for the buffer at a given wavelength (mdeg), c is the peptide concentration (mM), l is the path length (mm), and n is the number of amino acids^7^.

### Cell toxicity assays

The hemolytic activity of each peptide was evaluated using a modified method, as described previously^21^,^22^. Briefly, 1 mL of fresh hRBCs obtained from a healthy donor (Jiajun Wang, Harbin, China) in a polycarbonate tube containing heparin. The experimental protocol was reviewed and approved by the ethics committee of the Northeast Agricultural University Hospital, and the experimental method was carried out in accordance with the approved guidelines and regulations. Then, the collected hRBCs were washed three times with PBS (pH 7.2), centrifuged at 1000 × *g* for 5 min, and resuspended in PBS (final concentration, approximately 1%). Next, 50 μL of the hRBC suspension was placed into a 96-well cell culture plate with 50 μL of test peptide solution in PBS at different concentrations, and the mixture was incubated at 37 °C for 1 h. After incubation, the plate was centrifuged at 1000 × *g* for 5 min, the supernatant was transferred to a new 96-well cell culture plate, and the release of hemoglobin was measured by monitoring optical density (OD) at 576 nm. Untreated hRBCs suspension was used as a negative control, and the hRBCs treated with 0.1% Triton X-100 were employed as positive controls. Each test was reproduced at least 3 times using 6 replicates. Percent hemolysis was calculated using the following equation:


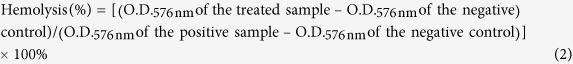


The MTT dye reduction assay was used to determine the cytotoxicity of each peptide on the murine macrophage cell line RAW264.7, according to a previously described method^21^. Briefly, 2.0 × 10^5^ cells well^−1^ in DMEM (supplemented with 10% heat-inactivated FBS) were placed into 96-well plates and then incubated for 24 h at 37 °C in 5% CO_2_. The next day, an increasing concentration of the peptides was added to cell cultures (1–128 μM), and untreated cell cultures served as controls. The cell cultures were further incubated for 24 h and then mixed with MTT (20 μL, 5 mgmL^−1^). After the mixtures were incubated for 4 h at 37 °C, they were centrifuged at 1,000 × *g* for 5 min, and the supernatants were discarded. Subsequently, 150 μL of DMSO was added to dissolve the formazan crystals formed, and the OD was determined at 570 nm using a microplate reader (TECAN GENiosF 129004; Tecan, Austria).

### Antimicrobial assays

Minimal bactericidal concentrations (MBCs) were measured using the method which has been adopted from National Committee for Clinical Laboratory Standards (NCCLS), with modifications^23^. In short, 50 μL peptide solutions were pipetted into microtiter plates at various concentrations ranging from 1 to 128 μM. Freshly grown bacteria were added to the each well in a density of 0.5–1 × 10^6^ CFUmL^−1^. Microtiter plated was incubated at 37 °C for 5 h. Fifty microliters of each incubation mixture was further serially diluted (up to 10^−3^ of the initial bacterial concentration), transferred on agar plates and incubated overnight. On the following day, the number of colonies was evaluated and the initial CFU/well retrospectively calculated. The minimum bactericidal concentrations (μM) were determined as the lowest concentration of the peptides that prevented more than 99.9% bacteria growth. Each test was reproduced at least 3 times using 6 replicates.

### Salt sensitivity assays

The salt sensitivity of the peptides was measured using a modified method, as described previously^24^. Bacterial cells were cultured in the presence of different concentrations of physiological salts to obtain the following final concentrations: 150 mM NaCl, 4.5 mM KCl, 2.5 mM CaCl_2_, 1 mM MgCl_2_, 8 μM ZnCl_2_, 6 μM NH_4_Cl, and 4 μM FeCl_3_. The subsequent steps were consistent with the MBCs determination method.

### Synergy with conventional antibiotics

The checkerboard titration method is commonly used to evaluate the synergy of different antibacterial combinations^25^. First, 2-fold serial dilutions of the antibiotics and antimicrobial peptides were prepared. Subsequently, 50 μL each of different concentrations of antibiotics and of antimicrobial peptides was mixed and added into 100 μL of bacterial solution (containing approximately 0.5–1 × 10^6^ CFU mL^−1^) in each well of a 96-well plate. The plates were then incubated at 37 °C for 24 h. The subsequent steps were consistent with the MBCs determination method. The fractional inhibitory concentration (FIC) index (FICI) was calculated as follows:


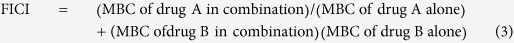


An FICI < 0.5 is considered to indicate synergy; 0.5 < FICI < 1.0 is considered additive; 1.0 < FICI < 4.0 is considered indifferent; and an FICI > 4.0 is considered antagonism.

### Dye leakage assays

Calcein-entrapped large unilamellar vesicles (LUVs) composed of POPC/POPG (3:1) were prepared as described previously^26^. Briefly, POPC/POPG (3:1) lipids were dissolved in chloroform, dried with a stream of nitrogen and resuspended in dye buffer solution (70 mM calcein, 10 mM Tris, 150 mM NaCl, 0.1 mM EDTA, pH 7.4). The suspension was subjected to 10 frozen–thaw cycles in liquid nitrogen and extruded 21 times through polycarbonate filters (two stacked 100-nm pore size filters) with a LiposoFast extruder (Avestin, Inc., Canada). Untrapped calcein was removed by gel filtration on a Sephadex G-50 column. Lipid concentration was determined by quantitative phosphorus analysis^27^. Aliquots of the liposome suspensions were diluted using Tris-HCl buffer to a final concentration of 100 μM lipid and incubated for 15 min with concentrations of peptide solution ranging from 1 to 128 μM. The leakage of calcein from LUVs was monitored by measuring fluorescence intensity at an excitation wavelength of 490 nm and emission wavelength of 520 nm on an F-4500 Fluorescence Spectrophotometer (Hitachi, Japan). The maximum dye leakage release obtained using 0.1% Triton X-100. The percentage of the calcein release caused by the AMPs was calculated using the equation:


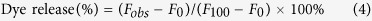


where *F*_*0*_ is the fluorescence intensity of liposomes (background), *F*_*obs*_ and *F*_*100*_ are intensities of the fluorescence achieved by peptides and Triton X-100, respectively.

### Forster Resonance Energy Transfer (FRET)

FRET measurements were performed to determine the strength of peptide association in different lipid membranes, POPG or POPC/POPG (3:1)^28^. Fluorescence spectra were measured by excitation at 492 nm with emission recorded in the range 500-700 nm using an F-4500 Fluorescence Spectrophotometer (Hitachi, Japan). Corrections for scattering were applied by subtracting a spectrum of a vesicle suspension lacking peptides. Total peptide concentration and FAM-labeled peptide concentration were kept constant so that the additive concentration of unlabeled and TAMRA-labeled peptide was constant although their respective ratio was not constant between samples. All samples were prepared in water and diluted with Tris-HCl (10 mM Tris, 150 mM NaCl, 0.1 mM EDTA, pH 7.4).

### Outer membrane permeability assays

The outer membrane permeabilization activity of peptides was determined by using the fluorescent dye NPN assay, as previously described^7^. Briefly, *E. coli* UB1005 cells were incubated to mid-log phase in MHB at 37 °C, harvested by centrifugation at 1,000 × *g* for 10 min, washed thrice, and diluted to 10^5^ CFUmL^−1^ with 5 mM HEPES buffer (pH 7.4, containing 5 mM glucose). Then, the cell suspensions were mixed with 10 μM NPN, and the background fluorescence was recorded for subtraction. (excitation λ = 350 nm, emission λ = 420 nm) with an F-4500 Fluorescence Spectrophotometer (Hitachi, Japan). Subsequently, 2 mL of cells suspension was added to a 1 cm quartz cuvette and mixed with different final concentrations of peptides. The fluorescence was recorded over time until no further increase in fluorescence was detectable. Values were converted to percent NPN uptake using the equation:


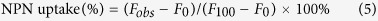


where *F*_*obs*_ is the observed fluorescence at a given peptide concentration, *F*_*0*_ is the initial fluorescence of NPN with *E. coli* UB1005 cells in the absence of peptide, and *F*_*100*_ is the fluorescence of NPN with *E*. *coli* UB1005 cells upon addition of 10 μg/mL polymyxin B (Sigma), which was used as a positive control because of its strong outer membrane-permeabilizing properties^7^.

### Cytoplasmic membrane depolarization assays

The cytoplasmic membrane depolarization activity of peptides was determined by using the membrane potential-sensitive fluorescent dye, diSC_3_-5 (Sigma-Aldrich), as previously described^24^. Briefly, *E. coli* UB1005 cells were incubated to the mid-log phase in MHB at 37 °C, harvested by centrifugation at 1,000 × *g* for 10 min, washed thrice, and diluted to an OD_600_ of 0.05 with 5 mM HEPES buffer (pH 7.4, containing 20 mM glucose) containing 0.1 M KCl to equilibrate the cytoplasmic and external K^+^. Subsequently, the cell suspensions were incubated with 0.4 μM diSC_3_-5 until maximal dye uptake was reached, and the background fluorescence was recorded for subtraction. (excitation λ = 622 nm, emission λ = 670 nm) with an F-4500 Fluorescence Spectrophotometer (Hitachi, Japan). Then, 2 mL of cell suspension was added to a 1 cm quartz cuvette and mixed with peptides used at their 0.5, 1, and 2 × MBCs. Changes in fluorescence were recorded from 0 to 600 s.

### Flow cytometry

The membrane damage was determined by flow cytometry^29^. Briefly, *E. coli* ATCC 25922 was grown to mid-log phase in MHB at 37 °C, harvested by centrifugation at 1,000 × *g* for 10 min, washed thrice with PBS and diluted to 10^5^ CFU mL^−1^ in the same buffer. The peptides at the concentrations of 1 × MBCs were incubated with the bacterial suspension at a fixed PI concentration of 10 μg mL^−1^ for 30 min at 4 °C, and followed by the removal of the unbound dye through washing with an excess of PBS. A Fluorescence Activated Cell Sorter (FACS) flow cytometer (Becton–Dickinson, USA) was used to obtain the data with a laser excitation wavelength of 488 nm.

### SEM and TEM characterizations

For SEM sample preparation, *E. coli* ATCC 25922 cells were cultured at 37 °C to mid-log phase in MHB under constant shaking at 220 rpm, harvested by centrifugation at 1,000 × *g* for 10 min, washed thrice, and diluted to an OD_600_ of 0.2 with 10 mM PBS. Cells were incubated for 60 min at 37 °C with different peptides used at their 1 × MBCs. The control cells were incubated without peptides. After incubation, the cells were harvested by centrifugation at 5,000 × *g* for 5 min, washed thrice with PBS, fixed with 2.5% (w/v) glutaraldehyde at 4 °C overnight, followed by washing twice with PBS (pH 7.2). The cells were dehydrated for 10 min with a graded ethanol series (50%, 70%, 90%, and 100%) and for 15 min in 100% ethanol. Then, the cells were transferred for 15 min in a mixture (v:v = 1:1) of 100% ethanol and *tert*-butanol and absolute *tert*-butanol. The specimens were dehydrated in a critical point dryer with liquid CO_2_, coated with gold-palladium, and then observed using a Hitachi S-4800 SEM.

Bacteria samples were initially prepared in the same way as for SEM. After pre-fixation with 2.5% glutaraldehyde at 4 °C overnight, the samples were washed twice with PBS, post-fixed with 2% osmium tetroxide for 70 min, and washed twice with PBS (pH 7.2). The bacteria samples were dehydrated for 8 min with a graded ethanol series (50%, 70%, 90%, and 100%) and for 10 min in 100% ethanol. Then, the cells were transferred for 10 min in a mixture (v:v = 1:1) of 100% ethanol and acetone and absolute acetone. The specimens were then immersed for 30 min in 1:1 mixtures of absolute acetone and epoxy resin. After centrifugation at 5,000 × *g* for 5 min, the specimens were incubated in pure epoxy resin overnight at a constant temperature. Then, the specimens were sectioned with an ultramicrotome, stained with uranyl acetate and lead citrate, and observed using a Hitachi H-7650 TEM.

### DNA-binding assays

Gel retardation experiments were performed, as previously described by Park *et al*. and Ulvatne *et al*.^8^,^30^,^31^. First, increasing concentrations of peptides were mixed with 400 ng of plasmid DNA (plasmid pUC57) in 20 μL of binding buffer (5% glycerol, 10 mM Tris-HCl (pH8.0), 1 mM EDTA, 1 mM dithiothreitol, 20 mM KCl, and 50 μgmL^−1^ bovine serum albumin). Next, the mixtures were incubated at 37 °C for 1 h. Subsequently, 4 μL of the native loading buffer (10% Ficoll 400, 10 mM Tris-HCl (pH 7.5), 50 mM EDTA, 0.25% bromophenol blue, and 0.25% xylene cyanol) was mixed with the samples, and a 12- μL aliquot was subjected to 1% agarose gel electrophoresis in 0.5 × Tris borate-EDTA buffer (45 mM Tris–borate and 1 mM EDTA, pH 8.0).

### Confocal laser scanning microscopy

To further analyze the intracellular target of the peptides *in vivo*, *E.coil* ATCC 25922 were incubated with PI-labeled peptides and observed on a confocal laser scanning microscopy. *E.coil* cells (OD_600_ = 0.2) were incubated with PI-labeled peptide at 1~2 × MBC at 37 °C. After incubation for 1 h, the cell pellets were collected by centrifugation at 5,000 × *g* for 5 min and washed three times with PBS buffer. A smear was made, and images were captured using a Leica TCS SP2 confocal laser scanning microscope with a 535 nm band pass filter for PI excitation.

### Statistical analysis

All data were submitted to one-way analysis of variance (ANOVA) and significant differences between means were evaluated by Tukey test for multiple comparisons. The data were analyzed using the Social Sciences (SPSS) version 20.0 (Chicago, Illinois, USA). Quantitative data were presented as the means ± standard error (SE). *P* < 0.05 was considered statistically significant.

## Results

### Peptide design

The centrosymmetric α-helical template was designed based on the α-helical protein framework and folding principles^32^. The peptide carboxyl O atom and amide proton between the I^th^ and (I + 4)^th^ amino acid positions form a paired hydrogen bond, resulting in a folded structure with a regular turn every 3.6 amino acids, and then the α-helix executes 97.2% of two turns in seven residues, so we arranged seven residues to comprise a primary centrosymmetric α-helical structure. Theoretical frameworks have demonstrated that hydrophobic residues tend to cluster in the n, n ± 3, and n ± 4 positions of adjacent helical turns that contribute toward the nucleation of this helical conformation and insertion into membranes^33^,^34^; therefore, by garnering hydrophobic interactions between the amino acid side groups at the 3^rd^ , 4^th^ and 5^th^ positions, we speculated that α-helical stabilization can be enhanced if the amino acids are not chosen from the helical breakers category^10^. Moreover, cationic repulsive forces prevent the α-helical formation of the peptide via the cationic amino acids at contiguous side chain positions^10^; therefore, the cationic amino acids were arranged at the 2^nd^ and 6^th^ positions. Amphipathicity accounts for α-helical AMP activity. Different AMPs exhibit amphipathicity with markedly varying hydrophobicity by maintaining well-defined and separate polar and hydrophobic motifs of varying sizes. Thus, for designing the template to be amphipathic when folded into α-helical structures, the amino acids of the 1^st^ and 7^th^ positions were also selected from hydrophobic amino acids, by converging the hydrophobic residues onto one side and the polar residues onto the other side of the helical axis^35^. However, some studies have reported that Gly residues are present with increased frequency at the 1^st^ and 7^th^ positions^17^ (e.g., >70% Gly in the 1^st^ position and >33% Gly in the 7^th^ positions); Gly is also an efficient N-capping agent which is known to influence the propensity of peptides to form α-helices^36^; therefore, Gly was tested at the 1^st^ and 7^th^ positions as well. In conclusion, the primary centrosymmetric α-helical sequence structure can be simplified as a repeat of the y + hhh + y amino acid sequence (y + hhh + y)_n_, where h represents a hydrophobic amino acid, +represents a cationic amino acid, and y represents a Gly or hydrophobic amino acid.

We constructed centrosymmetric peptides by utilizing the Antimicrobial Peptide Database (APD) and based on the primary centrosymmetric α-helical sequence template. Statistical analysis was conducted for a group of peptides from bacteria, plants, insects, and frogs, which were chosen because of a relatively large pool of peptides available in the APD^37^. It is notable that Gly residues are the most highly used amino acids (approximately 11.62%) in antimicrobial peptides from all the four kingdoms; this is followed by Lys (9.6%), Leu (8.62%), and Ala (7.71%). These four residues are incorporated with high frequency and also have a strong tendency to adopt an amphipathic helix^37^; therefore, we decided to utilize them to design peptides. Some researches have demonstrated that the middle position of the α-helical AMPs is important and has the greatest effect on peptide secondary structure and antimicrobial activity^38^. Consequently, to maximize the antimicrobial activity of centrosymmetric peptides, we selected Trp for the 4^th^ position, united with Leu at the 3^rd^ and 5^th^ position to constitute the active center of the AMPs, because the bulky side chain of Trp ensures more efficient interaction of peptides with the bacterial membrane^15^,^24^,^39^,^40^. Furthermore, Ala and Gly are small amino acids^19^. However, their α-helical properties are very different; Ala has the highest α-helical propensity of all amino acids^41^,^42^, but Gly allows for the most flexibility in the peptide chain, with the shortest side chain^17^. Thus, we designed the 1^st^ and 7^th^ positions with pairs of Ala and Gly, respectively, to study their different effects on the biological activity of peptides. Last, Lys is the only cationic amino acid that was arranged at the 2^nd^ and 6^th^positions.

We also designed some sequence scrambled peptides, in which the order of the amino acids was rearranged so that the sequences did not match centrosymmetric distribution. These sequence scrambled peptides had the same amino-acid composition as their synthetic counterparts and thus, the same molecular weight and charge. The sequences and naming scheme of all peptides are listed in Table 1.

### Peptide characterization

The molecular weight of peptide was verified by MALDI-TOF MS. Table 1 summarizes the theoretically calculated and measured molecular weights of each peptide. All peptides had molecular weight values consistent with their theoretical values, suggesting that the correct peptides were successfully synthesized. The hydrophobicity of the peptides in aqueous solution is reliably reflected by HPLC retention times. The centrosymmetric peptides presented varied HPLC retention times with the following hydrophobic order: AA4 > AA3 > AA2 > GG4 > GG3 > GG2 > AA1 > GG1, which was increased with an increase in chain length in each series. The HPLC retention times for sequence scrambled peptides GG3s1, GG3s2 and AA3s1 were 17.593, 16.994 and 23.131, respectively, which were similar with their respective synthetic counterparts.

The wheel diagram (Supplementary Fig. S2) and the relative hydrophobic moments (Table 1) revealed the amphipathy of all tested peptides. The results showed that the centrosymmetric peptides presented a better balance between the hydrophobic and hydrophilic phases compared with the sequence scrambled peptides.

### Secondary structure

**Figure 1:**
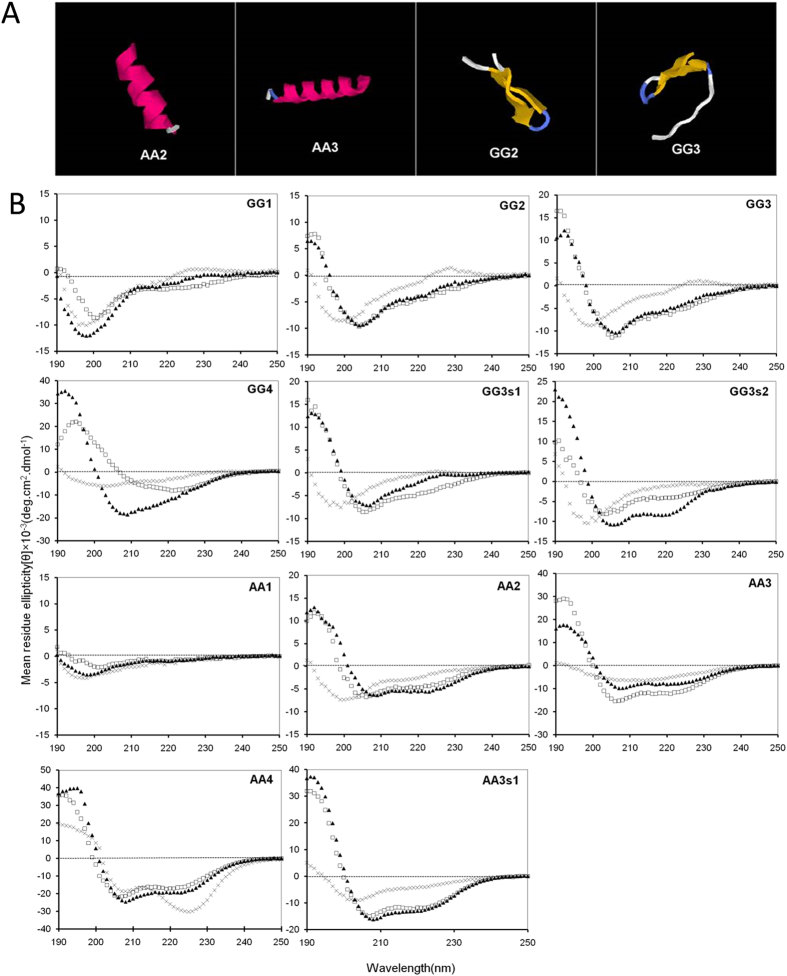
(**A**) Three-dimensional structure projections of AA2, AA3, GG2 and GG3. (**B**) The CD spectra of all peptides. All peptides were dissolved in 10 mM PBS (pH 7.4) (×), 50% TFE (□), or 30 mM SDS (▲). The mean residue ellipticity was plotted against wavelength. The values from three scans were averaged per sample, and the peptide concentrations were fixed at 150  μM.

The secondary structures of the peptides in membrane-mimetic environments were investigated by CD spectroscopy. The CD spectra (Fig. 1B) of the peptides were obtained at 150 μM under three conditions: 10 mM PBS (pH 7.4), 50% TFE, and 30 mM SDS. In 10 mM PBS, the spectra of all peptides except for AA4 were characteristic of unordered conformations. In membrane-mimetic environments, GG2 and GG3 showed a tendency to form helical structure, but the helical characteristics were negligible. In contrast, AA2 and AA3 displayed a typical α-helical spectrum, shown by the obvious negative ellipticity at around 208 and 222 nm, and exhibited higher α-helical content compared with GG2 and GG3, i.e. [θ]_222_ values of AA2(−5606) and AA3(−7868) compared with GG2(−3875) and GG3(-4921) in 30 mM SDS (Table 2). Moreover, in 30 mM SDS or 50% TFE, the helical content of the centrosymmetric peptides tended to increase with an increase in chain length, according to the following order: AA4 > AA3 > AA2 > AA1 and GG4 > GG3 > GG2 > GG1. The sequence scrambled peptides showed the similar capability to form α-helical structure in membrane-mimetic environments with their respective synthetic counterparts, but the only difference was that GG3s2 showed a typical α-helical structure in 30 mM SDS.

### Evaluation of toxicity

**Figure 2:**
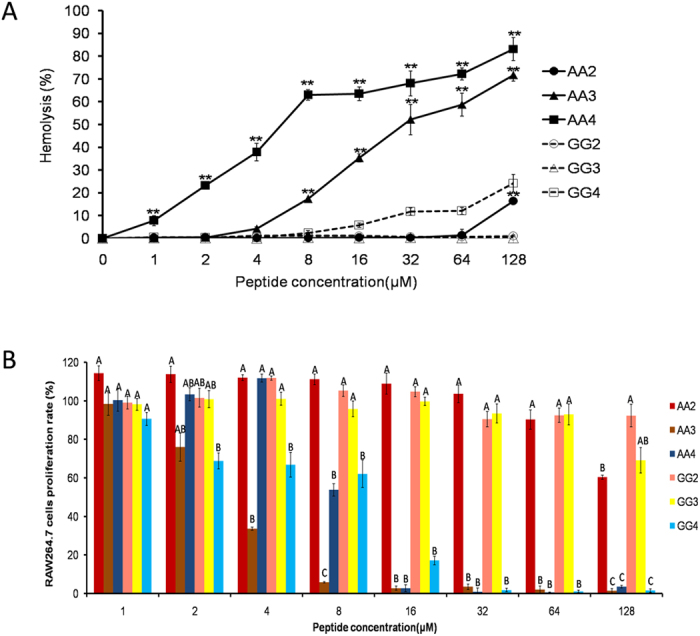
The security of the centrosymmetric peptides. (**A**) Hemolytic activity of the centrosymmetric peptides against hRBCs. (**B**) Cytotoxicity of the centrosymmetric peptides against RAW 264.7 cells. The graphs were derived from average values of three independent trials. P** < 0.01, compared to the values of GG2, GG3 and GG4, respectively at the same concentration. Means in the same concentration with different superscript are very significant difference (*P* < 0.01).

The toxicity of the peptides against hRBCs and RAW264.7 macrophage cells was evaluated at serial peptide concentrations in the range of 1–128 μM. It is desirable that GG2 and GG3 very significantly reduced hemolytic activity and cytotoxic activity compared with AA2 and AA3 (*P* < 0.01), respectively, at the highest concentration (Fig. 2). And as the same with GG3, GG3s1 and GG3s2 also kept weak cytotoxicity towards eukaryotic cells. Instead, the cell toxicity of AA3s1 was still high at 128 μM, but it was very significantly lowered compared with AA3 between 8 μM and 64 μM (*P* < 0.01) (Supplementary Fig. S3).

### Antimicrobial activity

The minimal bactericidal concentrations (MBCs) of all peptides are summarized in Table 3. As shown in results. The calculated geometric mean (GM) obtained by MBCs for all tested strains in the experiment, reflects the therapeutic effect of the peptides to typical bacterial strains in clinic. The GM values of the Gly-rich centrosymmetric peptides against the gram-negative strains were markedly lower than the gram-positive strains, exhibiting stronger antimicrobial activity against the gram-negative strains, yet Ala-rich centrosymmetric peptides displayed analogously effective antimicrobial activity toward the gram-negative and gram-positive strains. Compared with the GM values of the other centrosymmetric peptides, the centrosymmetric peptides designed with 3 repeat units showed the highest antimicrobial activity across the bacterial species, especially retaining the effective antimicrobial activity against the *Methicillin-resistant Staphylococcus aureus* ATCC 43300. The comprehensive GM values of centrosymmetric peptides across all bacterial species were according to the following order: A3 < G3 < A2 < A4 < G4 < G2. Meanwhile, the results also demonstrated that the antimicrobial activities of the sequence scrambled peptides were weaker than that of their respective synthetic counterparts, indicating that the centrosymmetric sequence did contribute to improve the antibacterial activity.

The therapeutic index (TI) is calculated by the ration of the minimum hemolytic concentration (MHC) to the geometric mean of the MBC (GM) to evaluate cell selectivity of peptides. Due to the great antimicrobial activity against gram-negative strains, the GG3 had the highest TI value against the gram-negative strains tested, at 101.59, an improvement of 10-fold compared to its TI value against the gram-positive strains tested. Thus, GG3 exhibited a considerably higher level of cell selectivity towards gram-negative bacteria cells over human red blood cells than others, implying a wider therapeutic window.

### Salt sensitivity assays

The antimicrobial activities of the centrosymmetric peptides were tested following the addition of physiological concentrations of different salts for the sensitivity assay (Table 4). Under these conditions, the MBC values for most of the centrosymmetric peptides did not vary obviously. Some cations, including mono valent (K^+^ and NH_4_^+^), divalent (Zn^2+^), and trivalent (Fe^3+^) cations, even exhibited a partial promoting effect on the antimicrobial activity of peptides at physiological concentrations. However, in the presence of Na^+^, GG2 and AA2 almost lost activity against *E. coli* ATCC 25922 and *S. aureus* ATCC 29213, respectively, while divalent cations (Mg^2+^ and Ca^2+^) also showed a weak inhibitory effect on the antimicrobial activity of peptides. In general, the centrosymmetric peptides with 3 and 4 repeat units were more tolerant of physiological salts than others.

### Synergy with conventional antibiotics

The antibacterial interactions between the centrosymmetric peptides and antibiotics were analyzed by the checkerboard assay. The FICI data for combinations of GG2, GG3, AA2, and AA3 are summarized in Table 5. GG3, AA2, and AA3 displayed synergism with streptomycin, with an FICI value of 0.5 against *E. coli*, whereas the peptides combinations with other antibiotics showed additive effects against *E. coli*; in all cases, FICI values from 0.75 to 1 were achieved. Although not all peptides had synergistic effects with antibiotics, the peptides usually showed an additive effect against *E. coli* with conventional antibiotics. Thus, the combination of peptides could substantially decrease the MBCs of the antibiotics (data not shown), indicating that the peptides are potential feed additives to reduce the antibiotic dose.

### Liposome leakage and FRET assays

Based on the MBCs and hemolytic values, Gly-rich centrosymmetric peptide GG3 displayed the best antimicrobial potential against gram-negative bacteria and the greatest cell selectivity, hence, GG3 and other centrosymmetric peptides as contrasts were chosen for further studying the antimicrobial mechanisms against the gram-negative bacteria.

**Figure 3:**
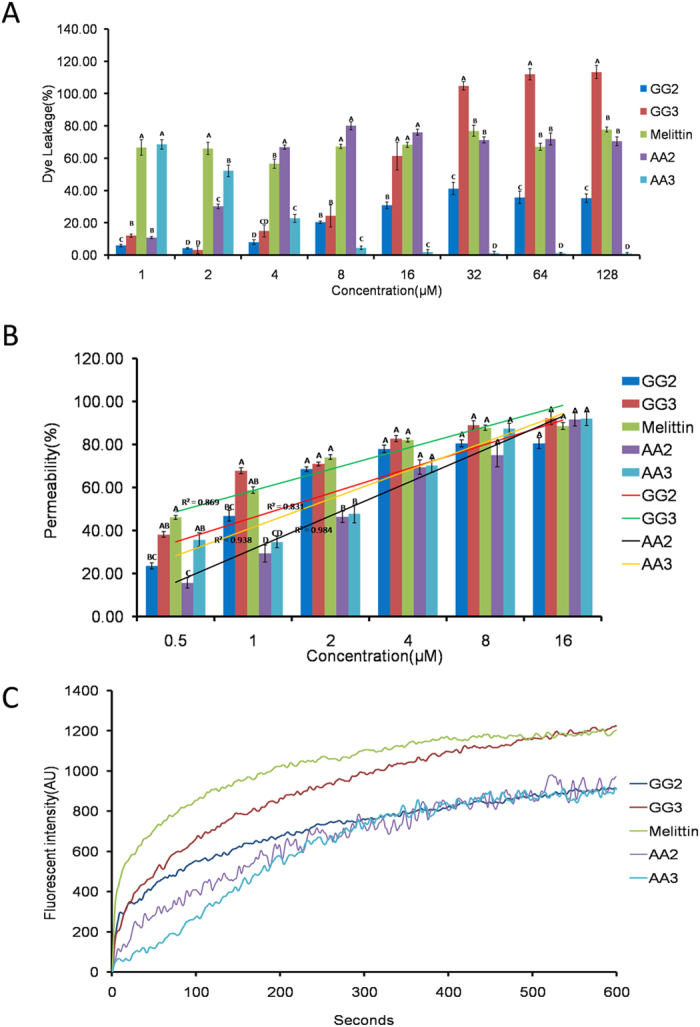
(**A**) Percent leakage of the dye calcein from negatively charged POPC/POPG (3:1) LUVs in the presence of the centrosymmetric peptides. (**B**) The outer membrane permeability of the centrosymmetric peptides, as determined using the fluorescent dye (NPN) assay. (**C**) The cytoplasmic membrane potential variation of *E. coli* UB1005 treated by 1 × MBC centrosymmetric peptides, as assessed by release of the membrane potential-sensitive dye diSC_3_-5. The graphs were derived from average values of three independent trials. Means in the same concentration with different superscript are very significant difference (*P* < 0.01).

Liposome leakage assay is usually conducted to test for pore formation. To evaluate whether the centrosymmetric peptides exert antimicrobial activities by pore formation and/or membrane perturbation, we measured their abilities to induce calcein leakage from negatively charged POPC/POPG (3:1) LUVs (bacterial membrane-mimicking environment). All the centrosymmetric peptides showed good leakage activity from negatively charged POPC/POPG (3:1) liposomes (Fig. 3A). Among them, GG3 exhibited a very significantly higher leakage activity than other centrosymmetric peptides above 16 μM (*P* < 0.01), indicating the destruction of liposomes by pore formation.

Furthermore, FRET experiment results showed that the values of *F/F*_*0*_ were very significantly negative linear correlated with the mole fraction *χ*_*A*_ of TAMRA-labeled GG3 (*P* < 0.01), with the R^2^ values of 0.779 and 0.840 in POPG or POPC/POPG (3:1) vesicles, respectively (Supplementary Fig. S4), further confirming the peptide-peptide interactions and/or aggregation of GG3 monomer in membrane-bound state, where the observed energy transfers between FAM-labeled GG3 / TAMRA-labeled GG3 in POPG or POPC/POPG (3:1) vesicles.

### Outer membrane permeabilization

The outer membrane plays an important role as a protective barrier in gram-negative bacteria. Thus, the ability of peptides to permeabilize the outer membrane was tested by using the NPN uptake assay. The hydrophobic fluorophore NPN is normally excluded from the outer membrane, but it is taken up and exhibits increased fluorescence intensity upon permeabilization of the outer membrane. All the centrosymmetric peptides were able to permeabilize the outer membrane of gram-negative bacteria at concentrations ranging from 0.5 to 16 μM, which followed a concentration-dependent increase in response (Fig. 3B). The outer membrane permeabilization caused by GG3 was very significantly higher than that caused by other centrosymmetric peptides in the presence of 1 μM peptide (*P* < 0.01), and above 4 μM, over 80% of outer membrane permeability of GG3 was measured, which was similar to that of melittin at the same concentration.

### Cytoplasmic membrane depolarization

The depolarization of the peptides on the *E. coli* cytoplasmic membrane was evaluated by using the membrane potential-sensitive dye diSC_3_-5. As shown in Fig. 3C, the increase in the relative fluorescence, reflecting membrane depolarization, was rapid induced by all centrosymmetric peptides, and GG3 caused more rapid and stronger membrane depolarization than the others over a period of 600 s, but it was weaker than the effect caused by melittin.

### Flow cytometry

**Figure 4:**
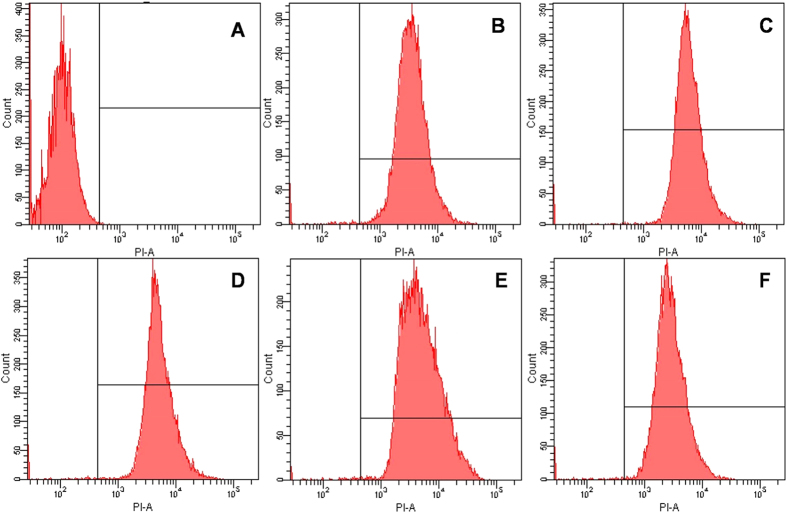
The membrane integrity damage of *E. coli* ATCC 25922 cells treated by the centrosymmetric peptides at 1 × MBC, as measured by an increase of fluorescent intensity of PI (10 μgmL^−1^) at 4 °C for 30 min. (**A**) No peptide (negative control, 0.1%); (**B**) GG2(98.8%); (**C**) GG3(99.2%); (**D**) AA2 (98.9%); (**E**) AA3 (99.3%); (**F**) Melittin (99.0%).

Some researches have pointed out that the ability of peptides to permeabilize the cell membrane does not mean that the cell membrane has been destroyed, more not equivalent to cell death^43^. Hence, these peptides were chosen for further studying the antimicrobial mechanisms by observing bacterial membrane integrity following peptides treatment. PI fluorescently stains the nucleic acids of cells indicating the disruption of cytoplasmic membrane integrity. It is a comprehensive indicator to measure the cell membrane integrity but does not distinguish between the cells' inner and outer membranes. The results demonstrated that the control (no peptide) only presented 0.1% PI fluorescent signal, indicating viable cell membranes (Fig. 4). However, when treated with peptides GG2, GG3, AA2 and AA3 at the concentration of 1 × MBC, the percentage of PI-positive cells increased to 98.8%, 99.2%, 98.9% and 99.3%, respectively, after 30 min of incubation. These results indicated that all tested centrosymmetric peptides killed the *E. coli* cell by destroying the cell cytoplasmic membrane integrity.

### SEM and TEM

**Figure 5:**
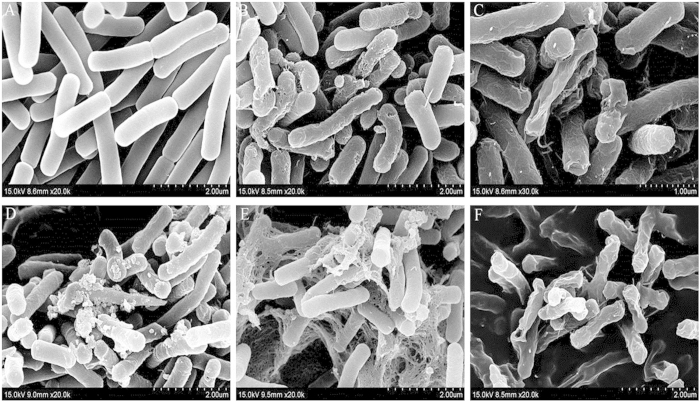
SEM micrographs of *E. coli* ATCC 25922: (**A**) Control; (**B**) GG2-treated; (**C**) GG3-treated; (**D**) AA2-treated; (**E**) AA3-treated; (**F**) Melittin-treated. Bacteria were treated with centrosymmetric peptides at 1 × MBC for 1 h. The control was processed without centrosymmetric peptides.

The cell morphology and membrane integrity upon treatment with peptides were directly observed by SEM (Fig. 5). Treatment with all centrosymmetric peptides at the 1 × MBCs for 1 h induced obvious membrane damage in comparison to the control, which exhibited a bright and smooth surface (Fig. 5A). The membrane surface of the *E. coli* cells treated with GG2 and GG3 became completely roughened and corrugated, with atrophy and fracture (Fig. 5B,C), similar to the cells treated with melittin (Fig. 5F). In comparison, the cell membrane surface treated with AA2 and AA3 showed more blebbing, and considerable intracellular content leakage was observed in the AA3-treated *E. coli* (Fig. 5D,E).

**Figure 6:**
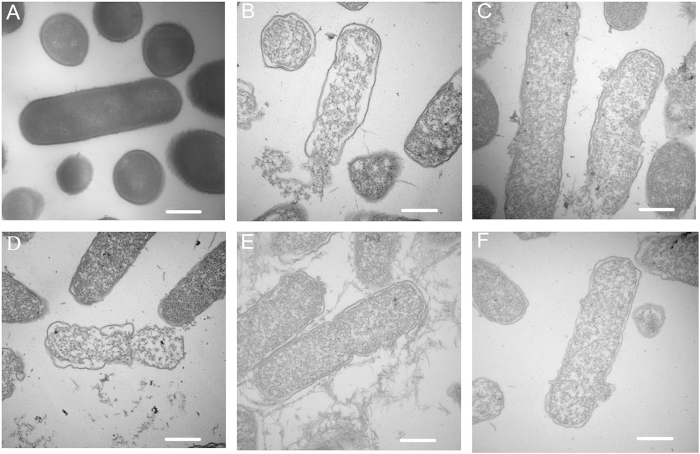
TEM micrographs of *E. coli* ATCC 25922: (**A**) Control; (**B**) GG2-treated; (**C**) GG3-treated; (**D**) AA2-treated; (**E**) AA3-treated; (**F**) Melittin-treated. Bacteria were treated with centrosymmetric peptides at 1 × MBC for 1 h. The control was processed without centrosymmetric peptides. Scale bar = 500 nm.

In addition to SEM, TEM was employed to study the morphology and intracellular alteration of *E. coli*, following treatment with centrosymmetric peptides. In comparison to the control, the treatment with the peptides induced obvious morphological changes in the membrane of and intracellular alterations in cells treated for 1 h with peptides used at 1 × MBCs (Fig. 6). After treatment with GG3 and AA3, visible pores with large diameters were observed, the cell membrane was disrupted, and the outer membrane separated from the inner membrane. Release of cellular content was also observed, which was similar to the response after treatment with melittin. The treatment with GG2 and AA2 induced more severe loss of membrane integrity and intracellular content; thus, the bacterial cells appeared to retain a cell shell.

### DNA binding

**Figure 7:**
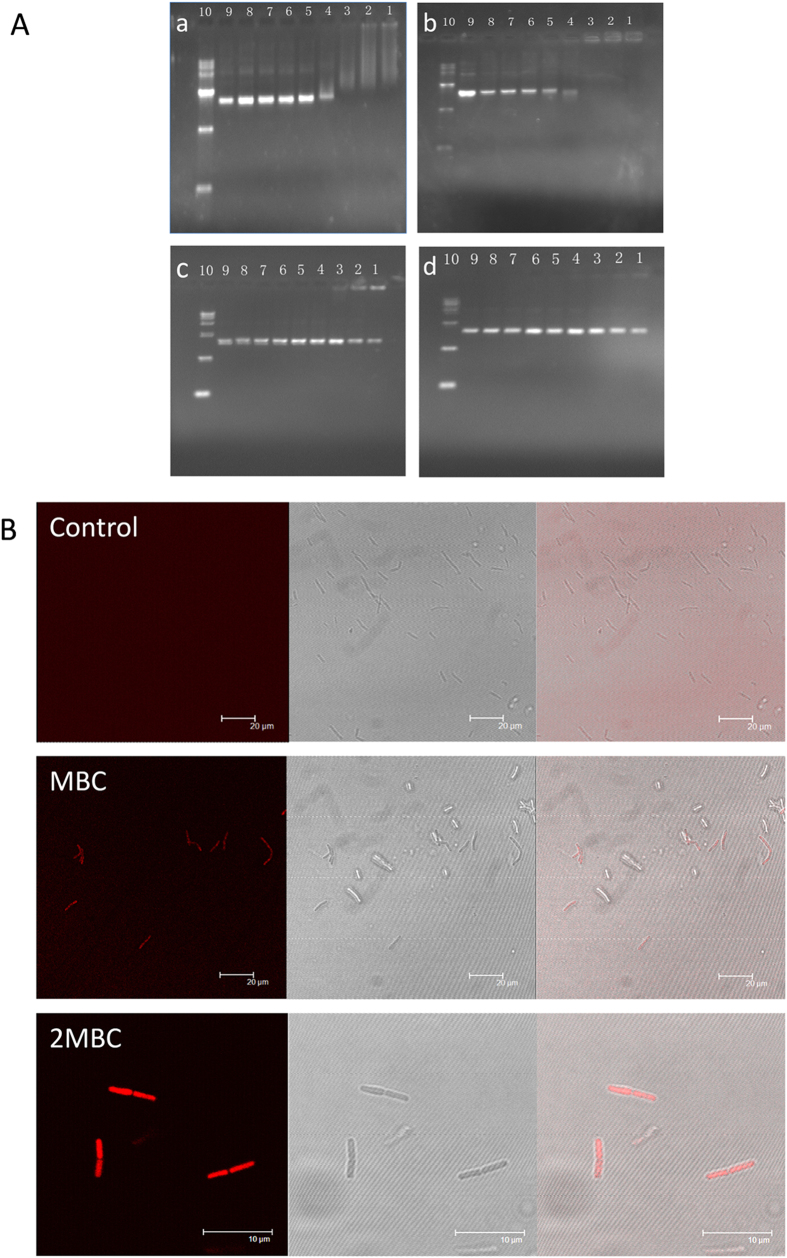
(**A**) DNA (pUC57) binding assay was determined by gel retardation experiments. Various concentrations of centrosymmetric peptides were incubated with 400 ng DNA for 1 h at room temperature. (**a**) GG2-treated; (**b**) GG3-treated; (**c**) AA2-treated; (**d**) AA3-treated; Lane 1: with 128 μM peptide; lane 2: with 64 μM peptide; lane 3: with 32 μM peptide; lane 4: with 16 μM peptide; lane 5: with 8 μM peptide; lane 6: with 4 μM peptide; lane 7: with 2 μM peptide; lane 8: with 1 μM peptide; lane 9: plasmid DNA alone; and lane 10: DNA Marker alone. (**B**) Confocal fluorescence microscopic images of *E. coil* ATCC25922 treated with PI-labeled GG3. Panels on the left, middle and right represent laser-scanning, transmitted light-scanning and merged images of cells treated with PI-labeled GG3, respectively.

AMPs can translocate to the cytoplasm and bind intracellular targets (e.g., DNA), leading to cellular inactivation. Thus, we investigated the possibility of intracellular effects, such as the DNA-binding properties of peptides. As shown in Fig. 7A, GG3 exhibited strong DNA-binding ability above 8 μM; In contrast, AA3 did not inhibit DNA migration even above 128 μM, which was possibly due to a lack of intracellular targets.

Moreover, to further investigate that GG3 also had the DNA-binding ability *in vivo*, the PI-labeled GG3 was incubated with log-phase *E. coli* ATCC 25922, and the binding ability of peptides was visualized by confocal laser scanning microscopy (Fig. 7B). The results showed that bacterial cells treated with PI-labeled GG3 appeared as red rod with fluorescence spread throughout the bacterial cell, indicating that PI-labeled GG3 molecules possessed ability to combine with DNA *in vivo*.

## Discussion

In this study, the centrosymmetric α-helical sequence template was designed with high amphipathicity by converging the hydrophobic residues onto one side and the polar residues onto the other side of the helical axis; therefore, all centrosymmetric peptides had an amphipathic residue arrangement with generally high relative hydrophobic moment values. Most previous studies reported that the conformational changes of amphipathic α-helical AMPs in various environments are important to biological activity^44^. Thus, the secondary structures of centrosymmetric peptides were measured by CD in the PBS and the membrane-mimetic environments (Fig. 1B). The results suggested that the type and number of amino acids in an AMP considerably influenced its secondary structure. Ala is the best helical promoter, thus, AA2 and AA3 displayed a typical α-helical structure in the membrane-mimetic environments, which was in accordance with the prediction (Fig. 1A). Nevertheless, Gly allows for the most flexibility in structure, because it is the amino acid with the shortest side chain; thus, the helical structures of GG2 and GG3 were not defined in comparison with Ala-rich centrosymmetric peptides, showing a slight negative peak at 222 nm in the CD spectra.

Hemolysis and cell toxicity are always considered the main obstacle for development of AMPs as further antimicrobial agent, so that they are the major parameters to evaluate the security of AMPs. As observed in our study, the hemolytic and cytotoxic activity of centrosymmetric peptides increased with an increase in hydrophobicity. The hemolysis and cytotoxicity of Gly-rich centrosymmetric peptides with 2 and 3 repeat units were very significantly lower than that of Ala-rich centrosymmetric peptides with 2 and 3 repeat units (*P* < 0.01), respectively, at the highest concentration, suggesting an improved selectivity of Gly-rich centrosymmetric peptides with 2 and 3 repeat units towards the anionic component of microbial cell membranes over zwitterionic mammalian cell membranes, presumably due to the rational hydrophobicity and α-helical propensity of these peptides^8^. It is worth noting that the hemolysis and cytotoxicity are very significantly positive correlated with hydrophobicity and α-helical propensity (*P* < 0.01) (Supplementary Fig. S5), which similarly improve antimicrobial activity. Therefore, a suitable balance between hydrophobicity and α-helical propensity is required to achieve the optimal biological activity^5^,^11^,^45^.

The antimicrobial assays demonstrated that the centrosymmetric peptides with 3 repeat units possessed the highest activity of the centrosymmetric peptides. This result was most likely due to the increases in the net positive charge. As observed in previous reports, an increase in cationic charge is clearly important in the initial interaction of AMPs with the anionic microbial surfaces^46^. However, in the present study, we found that the antimicrobial activity of peptides with +7 charges was the strongest among all peptides, but with excessive charge density was not further improved. Thus, the relationship between the cationicity and antimicrobial activity is not simple positive correlation and presents a threshold (Supplementary Fig. S6). The result is consistent with previous studies that +6 ~+ 7 charge on the peptides may be optimal and higher cationicity instead compromises activity^47^; excessive charge density on the membrane can inhibit the peptide packing on the vesicle surface and penetration of the membrane^48^. Furthermore, the relationship between hydrophobicity and biological activity similarly presents a threshold, such that increasing the hydrophobicity above the threshold makes the molecule too hydrophobic to retain antibacterial activity^49^. However, the hydrophobicity of AA2 and GG4 were in between AA3 and GG3, suggesting that the cationicity is more preferential than hydrophobicity for antimicrobial activity, possibly due to it determining the initial electrostatic attraction of peptides to the membrane.

Amphipathicity and α-helix propensity also appear to have influence on the antimicrobial activity of the centrosymmetric peptides. Amphipathic residue arrangement is important for the activity of α-helical AMPs because the polar region allows the molecules to assemble on the membrane through electrostatic interaction with the negatively charged head groups of phospholipids, and then the nonpolar region would lead to the formation of transient pores or channels through hydrophobic interactions, causing increased permeability and loss of barrier function of target cells^34^. If the peptides display helix formation in the membrane environment, they would have greater affinity for the anionic membrane and have enhanced incorporation into lipid membranes, further increasing the antimicrobial activity of AMPs^50^,^51^. Based on the CD spectra and wheel diagram results, the Ala-rich centrosymmetric peptides had the potential to form a more typical α-helical structure along with ideal amphipathy than Gly-rich centrosymmetric peptides, which was consistent with their relatively stronger broad-spectrum antimicrobial activity, but also with higher cytotoxicity. Thus, amphipathicity and α-helical propensity have dual characters, a delicate balance of amphipathicity and α-helical propensity is necessary. For maintaining antimicrobial activity with low hemolytic activity, Gly was introduced to replace Ala in the nonpolar face. As observed in previous reports, the hydrophobicity of Gly is slightly lesser than that of Ala^52^, and peptide amphipathicity is directly positive correlated with side chain hydrophobicity of the amino acid residue for the nonpolar face^38^. Additionally, Gly is compatible with helical conformation in membrane proteins, but, while it has a considerable tendency to form α-helices under membrane mimetic conditions, Gly is somewhat destabilizing for the α-helical structure compared to the more hydrophobic side chains^17^,^19^. Hence, compared to Ala-rich centrosymmetric peptides, Gly-rich centrosymmetric peptides exhibited slightly lower amphipathicity and obviously weaker α-helix propensity, yet they did not markedly reduce activity toward gram-negative bacteria, while dramatically increasing selectivity. This finding is consistent with a previous study that demonstrated that reduction in the stability of helix formation with glycine may quite effectively narrow the activity spectrum to a subset of the gram-negative species^11^. Furthermore, extensive characterization showed that the double membrane of gram-negative bacteria is a less efficient barrier than the single membrane and the thick peptidoglycan layer of the gram-positive bacteria; consequently, gram-negative bacteria is more susceptible to permeabilization than gram-positive bacteria^11^. Thus, as mentioned above, we have come to the conclusion that the Gly-rich centrosymmetric peptides GG3 with poorly helical structure, showed great specific selectivity for gram-negative bacteria, while exhibiting an optimal balance between antimicrobial activity and hemolytic activity.

It is interesting to note that physiological concentrations of salts would slightly compromise the antimicrobial activity of the centrosymmetric peptides. According to the mechanism of action proposed, we know that the electrostatic interactions between peptides and bacteria membrane are the premise for peptides to exert antimicrobial activity. Therefore, it is not surprising to observe that the charge screening effects by Na^+^ and other cations can hinder the electrostatic interaction^24^,^50^, which prevent peptides from binding the bacterial membrane and results in decreased AMPs antimicrobial activity. Not only by that, the divalent (Ca^2+^ and Mg^2+^) cations affect the activity of AMPs but also by the competition between the peptides and cations for interactions with the LPS in membrane binding^53^. And excess divalent cations progressively increase membrane rigidity through the electrostatic interactions with negatively charged phospholipids, and this effect slowly hinders pore formation^54^. However, this is not always the case, some reports also demonstrated that additional net positive charge may decrease the effect of cations on the electrostatic interactions^7^, and this is consistent with our results that the centrosymmetric peptides with 3 and 4 repeat units were more tolerant of physiological salts than others. Thus, the centrosymmetric peptides with 3 and 4 repeat units with higher net positive charges had greater affinities for the bacterial membrane and overcame the adverse effects of cations, thus maintaining the good antimicrobial activity in the presence of salts

Previous studies have reported that synergistic effects among conventional antibiotics and peptides can improve the antibacterial activity^8^,^55^. This effect is attributed to the enhanced intracellular access of the drug aided by these membrane-permeabilizing peptides^25^. In our study, GG3, AA2, and AA3 displayed synergism with streptomycin and showed additive effects with other antibiotics against *E. coli*. However, GG2 had no interaction with ceftazidime and ciprofloxacin, in part because it had lower membrane permeabilization than other peptides. Although not all centrosymmetric peptides had synergistic effects with antibiotics, the peptides could substantially decrease the MBCs of the antibiotics, indicating that the centrosymmetric peptides are potential feed additives for reducing the dose of antibiotics.

As indicated by previous reports, α-helix conformation plays a crucial role in determining the activity of AMPs, which indicates that the peptides exert antimicrobial activity via membrane destruction^56^. However in our study, GG3 with the poorly α-helical propensity, stunningly exhibited the strongest activity to permeabilize the outer membrane, dissipate the transmembrane electrochemical gradient and induce pore formation, which would facilitate cell cytoplasmic leakage and lead to cellular inactivation. Furthermore, flow cytometry, SEM and TEM results further confirmed that GG3 killed bacteria via membrane destruction and content leakage. Thus, a possible explanation for those may be that the Gly-Gly pairs motif contributes to the membrane disruption. The small amino acid distribution pattern (i.e., small motifs, particularly Gly-Gly pairs) is an important factor increasing peptides activity^57^. The motif of Gly-Gly pairs is implicated in the promotion of helix-helix interactions in the membrane^19^, which have dual role of facilitating the passage of unstructured molecules through cell wall components^58^, and of increasing the probability of transient monomer aggregation and pore formation in the cytoplasmic membrane^57^, all of which would make up for the poorly α-helical propensity and contribute to the membrane destruction ability of the centrosymmetric peptides. Simultaneously, it further explains the results that GG3 maintained the great activity against gram-negative bacteria. In addition, there is increasing evidence indicating that AMPs can translocate to the cytoplasm to bind intracellular targets^2^ (i.e., DNA), altering the cytoplasmic membrane septum formation, inhibiting cell-wall, nucleic acid, and protein syntheses, or inhibiting enzymatic activity, eventually leading to cellular inactivation^59^. Unlike AA3, GG3 adopted a poorly helical structure in membrane-mimetic environments, but bound to a short duplex DNA sequence with high affinity *in vitro* and *in vivo*. This results showed that the α-helical structure was not required for the DNA binding activity^60^. Collectively, GG3 exerted its antimicrobial activity by damaging the integrated cell membrane by pore formation, inducing the membrane atrophy and fracture due to leakage of the intracellular contents, and further exerted antimicrobial activity by traversing the bacterial membrane and binding DNA.

## Conclusion

In this study, we established a centrosymmetric α-helical sequence template based on the α-helical protein-folding principles to guide the peptides design with pairs of small amino acids. This method which maintains evolutionarily conserved sequence patterns enhances the antimicrobial activity of peptides, while reducing the number of the candidate AMPs required to obtain useful results. Different lengths and subtypes of hydrophobic amino acids considerably influenced the helical structure of the peptides under membrane-mimetic conditions, while affecting the antimicrobial activity and hemolytic activity of the peptides. The Gly-rich centrosymmetric peptide with 3 repeat units (GG3) possessed the poorly helical structure, but still exhibited better selectivity for gram-negative bacteria without hemolysis, indicating that reduction in the stability of helix formation with glycine improved the specific selectivity of peptides for gram-negative bacteria. Furthermore, GG3 also showed strong high salt tolerance and obvious synergy with conventional antibiotics. GG3 exerted its bactericidal effects by damaging the membrane integrity, leading to leakage of the cytosol and eventual cell lysis at its MBC, possibly due to Gly-Gly pairs which contribute to the membrane destruction ability. Additionally, GG3 further exerted antimicrobial activity by binding DNA, suggesting that the α-helical structure was not required for the DNA binding activity. All these findings demonstrated that the Gly-rich centrosymmetric peptide GG3 was a promising antimicrobial agent against gram-negative bacteria. And the template-assisted design was a potent approach for expanding the arsenal of the AMP families. Although the results reported here reflect the *in vitro* situation and at an early stage, the Gly-rich centrosymmetric peptide GG3 will be further assessed *in vivo* situation with the ultimate purpose of development of antimicrobial agents.

## Additional Information

**How to cite this article**: Wang, J. *et al*. High specific selectivity and Membrane-Active Mechanism of the synthetic centrosymmetric α-helical peptides with Gly-Gly pairs. *Sci. Rep*. **5**, 15963; doi: 10.1038/srep15963 (2015).

## Supplementary Material

Supplementary Information

## Figures and Tables

**Table 1:** Peptide design and their key physicochemical parameters.

	Peptide	Sequence	Theoretical MW	Measured MW^a^	Net charge	Retention time (min)	μHrel^b^
Gly-rich centrosymmetric peptides	GG1	GKLWLKG-NH2	800.00	800.02	3	13.505	0.503
	GG2	GKLWLKGGKLWLKG-NH2	1582.98	1583.01	5	16.053	0.495
	GG3	GKLWLKGGKLWLKGGKLWLKG-NH2	2365.97	2366.01	7	17.282	0.483
	GG4	GKLWLKGGKLWLKGGKLWLKGGKLWLKG-NH2	3148.95	3149.00	9	18.572	0.465
Ala-rich centrosymmetric peptides	AA1	AKLWLKA-NH2	828.05	828.08	3	13.997	0.550
	AA2	AKLWLKA AKLWLKA-NH2	1639.09	1639.12	5	18.581	0.539
	AA3	AKLWLKAAKLWLKAAKLWLKA -NH2	2450.13	2450.17	7	24.655	0.525
	AA4	AKLWLKAAKLWLKAAKLWLKAAKLWLKA-NH2	3261.17	3261.21	9	28.023	0.506
sequence scrambled peptides	GG3s1	GKLWGLKGKLWGLKGKLWGLK -NH2	2365.97	2366.01	7	17.593	0.262
	AA3s1	AKLWALKAKLWALKAKLWALK -NH2	2450.13	2450.17	7	23.131	0.201
	GG3s2	GLWKGKLGLWKGKLGLWKGKL -NH2	2365.97	2366.01	7	16.994	0.414

^a^Molecular weight (MW) was measured by mass spectroscopy (MS).

^b^The relative hydrophobic moment (μHrel) of a peptide is its hydrophobic moment relative to that of a perfectly amphipathic peptide. This gives a better idea of the amphipathicity using different scales. A value of 0.5 thus indicates that the peptide has about 50% of the maximum possible amphipathicity.

**Table 2:** CD data of the peptides in various solutions.

Peptides^a^	PBS		SDS		TFE	
	[θ]_222_^b^	%helix^c^	[θ]_222_	%helix	[θ]_222_	%helix
GG1	−470	3	−1976	12	−2953	18
GG2	−552	3	−3875	23	−4388	27
GG3	−609	4	−4921	30	−5600	34
GG4	−2667	16	−11432	69	−8016	48
AA1	−1344	8	−739	4	−566	3
AA2	−2234	14	−5606	34	−4323	26
AA3	−4815	29	−7868	48	−11588	70
AA4	−28590	173	−19310	117	−16536	100
GG3s1	−41	0	−1411	9	−4398	27
AA3s1	−4069	25	−12598	76	−11842	72
GG3s2	−995	6	−7841	47	−4032	24

^a^The peptides were dissolved in aqueous (10 mM sodium phosphate buffer, PH 7.4) or membrane-mimicking solvent (50% TFE or 30 mM SDS micelles).

^b^The mean residue molar ellipticities [θ]_222_ (degree.cm^2.^dmol^−1^) at wavelength 222 nm were measured at 25˚C.

^c^The helical content (%) of a peptide relative to the molar ellipticity value of peptide AA4 in 50% TFE.

**Table 3:** MBCs, MHC and therapeutic indices of all peptides.

	GG1	GG2	GG3	GG3s1	GG3s2	GG4	AA1	AA2	AA3	AA3s1	AA4	Melittin
Minimum bactericidal concentrations (μM)^a^												
Gram-negative bacteria												
*E. coli 25922*	>128	4	2	4	4	4	>128	1	2	2	8	2
*E. coli 1005*	>128	32	2	4	4	8	>128	8	2	4	32	2
*S. typhimurium 14028*	>128	64	4	16	32	32	>128	16	4	16	128	2
Gram-positive bacteria												
*S. aureus 29213*	>128	128	16	128	128	128	>128	16	4	16	32	8
*S. aureus 43300*	>128	64	32	128	64	128	>128	16	4	16	32	1
*S. epidermidis 12228*	>128	128	32	128	64	>128	>128	32	4	64	32	1
GM (geometric mean of MBCs)^b^												
*Gram* (−)^I^	>128	20.16	2.52	6.35	8	10.08	>128	5.04	2.52	5.04	32	2
*Gram* (*+*)^II^	>128	101.59	25.40	128	80.63	161.27	>128	20.16	4	25.40	32	2
*Gram* (*+*, *−*)^III^	>128	45.25	8	28.51	25.40	40.32	>128	10.08	3.17	11.31	32	2
*MHC*(*μM*)^c^	>128	>128	>128	>128	>128	16	>128	128	8	16	1	0.25
TI(Therapeutic index)^d^												
*TI(-)*I	n.a.^e^	12.70	101.59	40.31	32	1.59	n.a.	25.40	3.17	3.17	0.03	0.13
*TI*(*+*)^II^	n.a.	2.52	10.08	2	3.17	0.10	n.a.	6.35	2	0.63	0.03	0.13
*TI*(*+*, *−*)^III^	n.a.	5.66	32	8.98	10.08	0.40	n.a.	12.70	2.52	1.41	0.03	0.13

^a^Minimum bactericidal concentrations (μM) were determined as the lowest concentration of the peptides that prevent more than 99.9% bacteria growth. The data were derived from representative value of three independent experimental trials.

^b^The geometric mean (GM) of the peptide MBCs against all bacterial strains was calculated. When no detectable antimicrobial activity was observed at 128 μM, a value of 256 μM was used to calculate the therapeutic index. (I): against 3 gram-negative strains tested; (II): against 3 gram-positive strains tested; (III): against all of the 6 strains tested.

^c^MHC is the minimum hemolytic concentration that caused 5% hemolysis of human red blood cells. When no detectable hemolytic activity was observed at 128 μM, a value of 256 μM was used to calculate the therapeutic index.

^d^TI is calculated as MHC/GM. Larger values indicate greater cell selectivity.

^e^Not available due to little bioactivity.

**Table 4:** MBC values of centrosymmetric peptides in the presence of physiological salts.

Peptide	Control^a^	NaCl^a^	KCl^a^	NH_4_Cl^a^	MgCl_2_^a^	CaCl_2_^a^	ZnCl_2_^a^	FeCl_3_^a^
*Gram-negative strain E. coli* ATCC 25922								
GG1	>128	>128	>128	>128	>128	>128	>128	>128
GG2	4	>128	4	4	16	16	4	4
GG3	2	2	2	2	2	2	2	1
GG4	4	4	4	4	4	4	4	4
AA1	>128	>128	>128	>128	>128	>128	>128	>128
AA2	1	2	1	1	2	4	1	1
AA3	2	2	1	2	2	2	1	2
AA4	8	64	4	8	8	8	4	8
*Gram-positive strain S. aureus* ATCC 29213								
GG1	>128	>128	>128	>128	>128	>128	>128	>128
GG2	128	>128	128	128	>128	>128	128	128
GG3	16	16	8	16	16	16	16	16
GG4	128	>128	128	128	>128	128	128	128
AA1	>128	>128	>128	>128	>128	>128	>128	>128
AA2	16	128	16	8	32	64	8	16
AA3	4	4	4	4	4	4	4	4
AA4	32	32	32	32	32	128	32	32

^a^The final concentrations of NaCl, KCl, NH_4_Cl, MgCl_2_, CaCl_2_, ZnCl_2_, and FeCl_3_ were 150 mM, 4.5 mM, 6 μM, 1 mM, 2 mM, 8 μM, and 4 μM, respectively, and the control MBC values were determined in the absence of these physiological salts. The data were derived from representative value of three independent experimental trials.

**Table 5:** The FIC indexes^a^ for the centrosymmetric peptides in combination with antibiotics against *E. coli* ATCC 25922.

Peptide	Ceftazidime^b^	Chloramphenicol^b^	Streptomycin^b^	Ciprofloxacin^b^
GG2	2	1	1	2
GG3	0.75	1	0.5	1
AA2	1	1	0.5	0.75
AA3	1	1	0.5	2

^a^FIC index < 0.5 as synergy, 0.5 < FICI < 1.0 as additive, 1.0 < FICI < 4.0 as indifferent, and FICI > 4.0 as antagonism.

^b^The MBC values of Ceftazidime, Chloramphenicol, Streptomycin, Ciprofloxacin were 2 μM, 8 μM, 2 μM, 16 μM, respectively against *E. coli* ATCC 25922.
