# Supplementary material for: High specific selectivity and Membrane-Active Mechanism of the synthetic centrosymmetric α-helical peptides with Gly-Gly pairs
Source: Sci Rep. 2015 Nov 4;5:15963. doi: 10.1038/srep15963 (PMC4632126; doi:10.1038/srep15963)
Supplement: Supplementary Information [file srep15963-s1.doc]

**Supporting information**

**High specific selectivity and Membrane-Active Mechanism of the synthetic centrosymmetric α-helical peptides with Gly-Gly pairs**

**Jiajun Wang, Shuli Chou, Lin Xu, Xin Zhu, Na Dong, Anshan Shan*, Zhihui Chen**

Institute of Animal Nutrition, Northeast Agricultural University, Harbin 150030, P. R. China


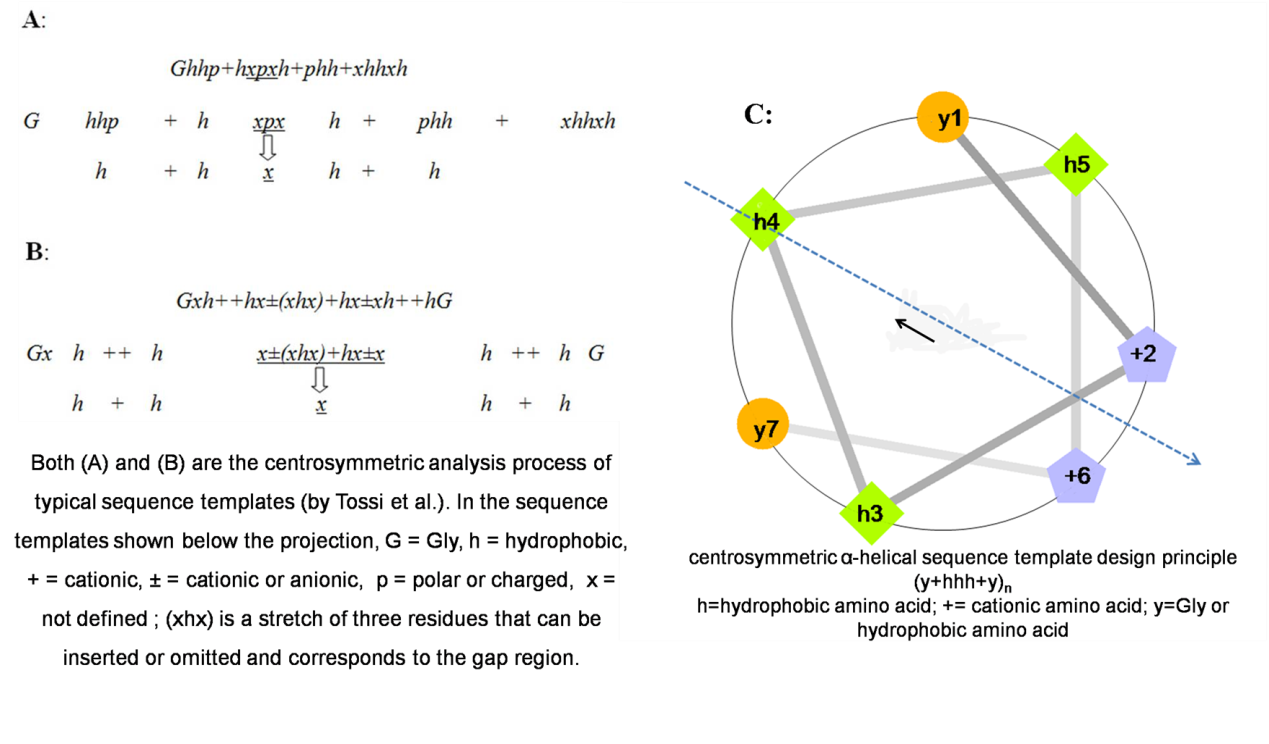


Supplementary Fig. S1. Both (A) and (B) are the centrosymmetric analysis process of typical sequence templates (by Tossi et al.). In the sequence templates shown below the projection, G = Gly, h = hydrophobic, + = cationic, ± = cationic or anionic, p = polar or charged, x = not defined ; (xhx) is a stretch of three residues that can be inserted or omitted and corresponds to the gap region;

(C) Centrosymmetric α-helical sequence template design principle (y+hhh+y)_n_ h=hydrophobic amino acid; += cationic amino acid; y=Gly or hydrophobic amino acid.


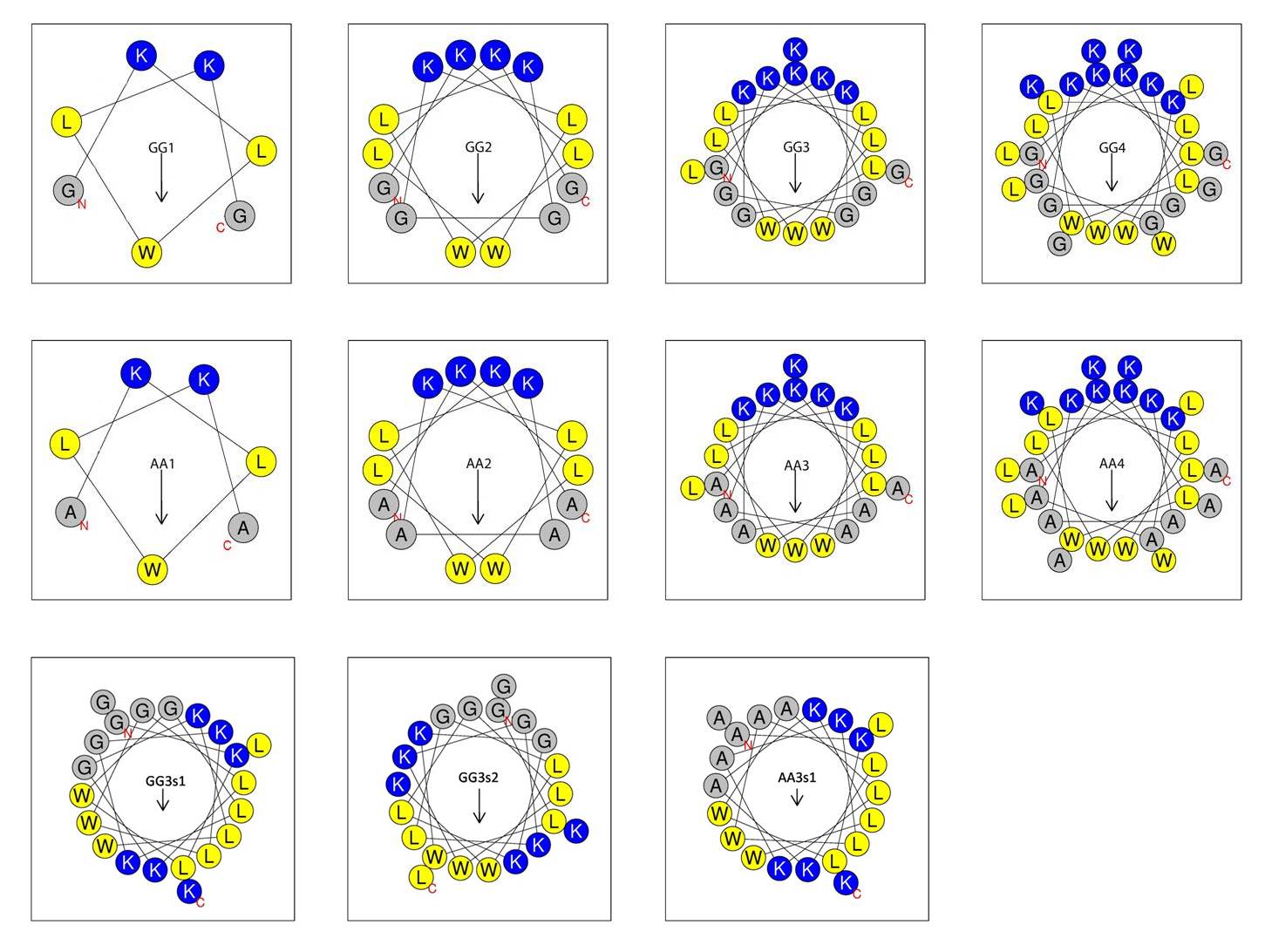


Supplementary Fig. S2. Helical wheel projections of the peptides. Light blue and yellow circles represent hydrophilic and hydrophobic residues, respectively. The grey represents the small amino acids.

B


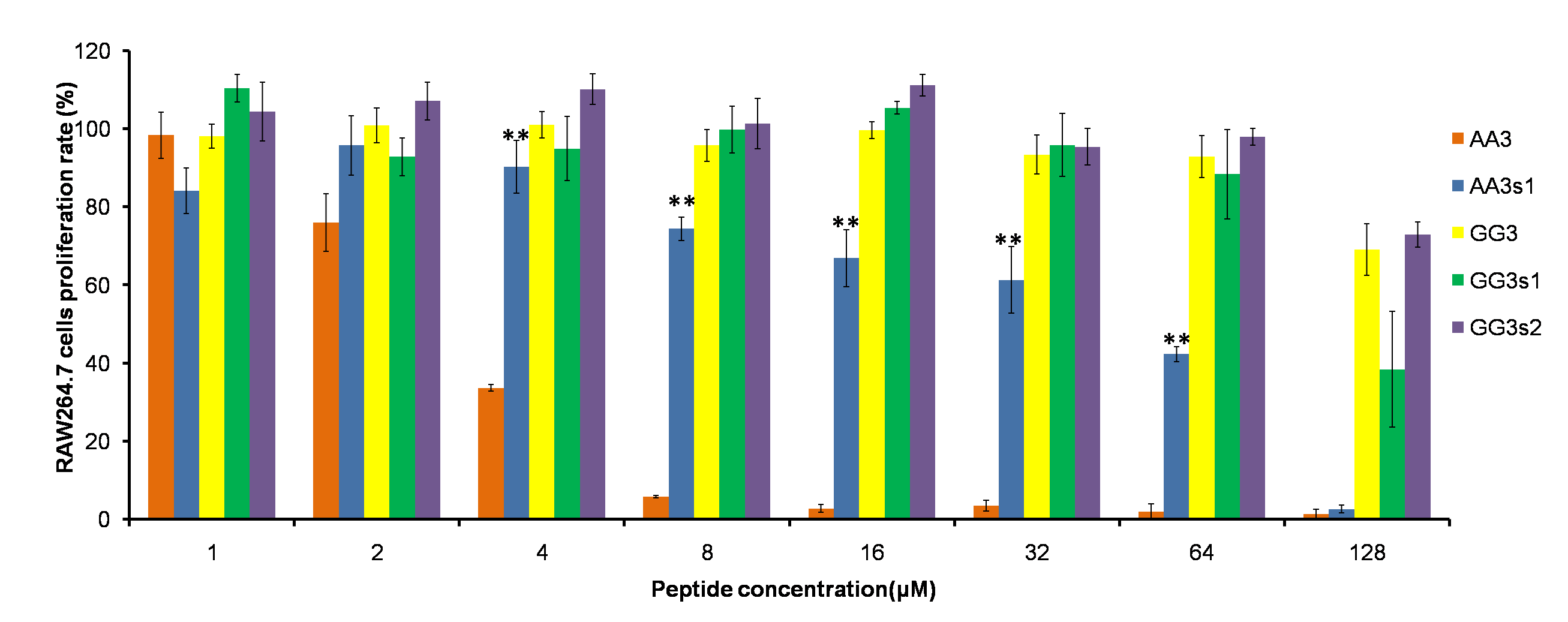

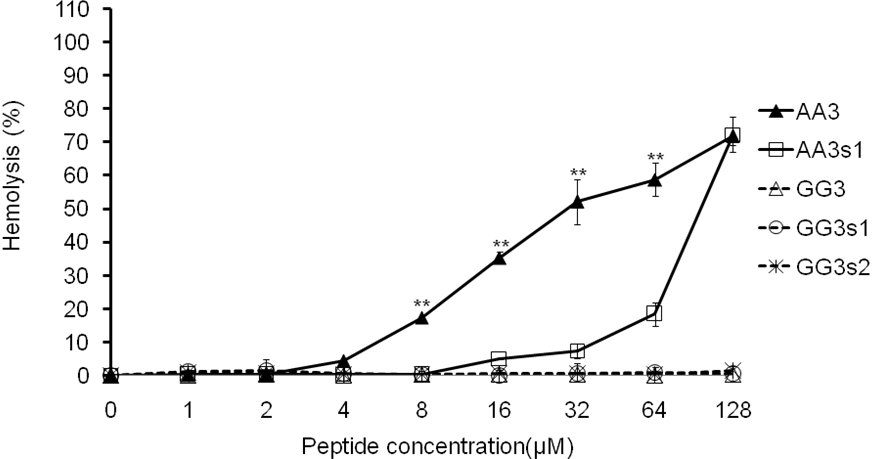


A

Supplementary Fig. S3. The security of the sequence scrambled peptides. (A) Hemolytic activity of the sequence scrambled peptides against hRBCs. (B) Cytotoxicity of the sequence scrambled peptides against RAW 264.7 cells. The graphs were derived from average values of three independent trials. *P**<*0.01, compared to the values of their respective counterparts at the same concentration.


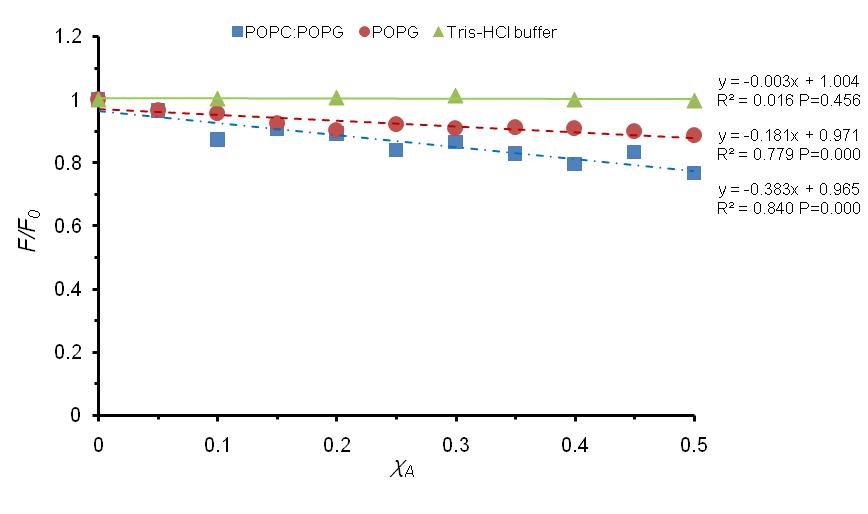


Supplementary Fig. S4. Association of GG3 as determined by FRET. The ratio of fluorescence intensity in the presence (*F*) and absence (*F_0_*) of acceptor was measured as a function of the mole fraction *χ_A_* of TAMRA-labeled peptides. The mole fraction of TAMRA-labeled peptide*χ_A_* was varied in the range 0-0.5, while the total peptide concentration was kept constant by using unlabeled peptides.


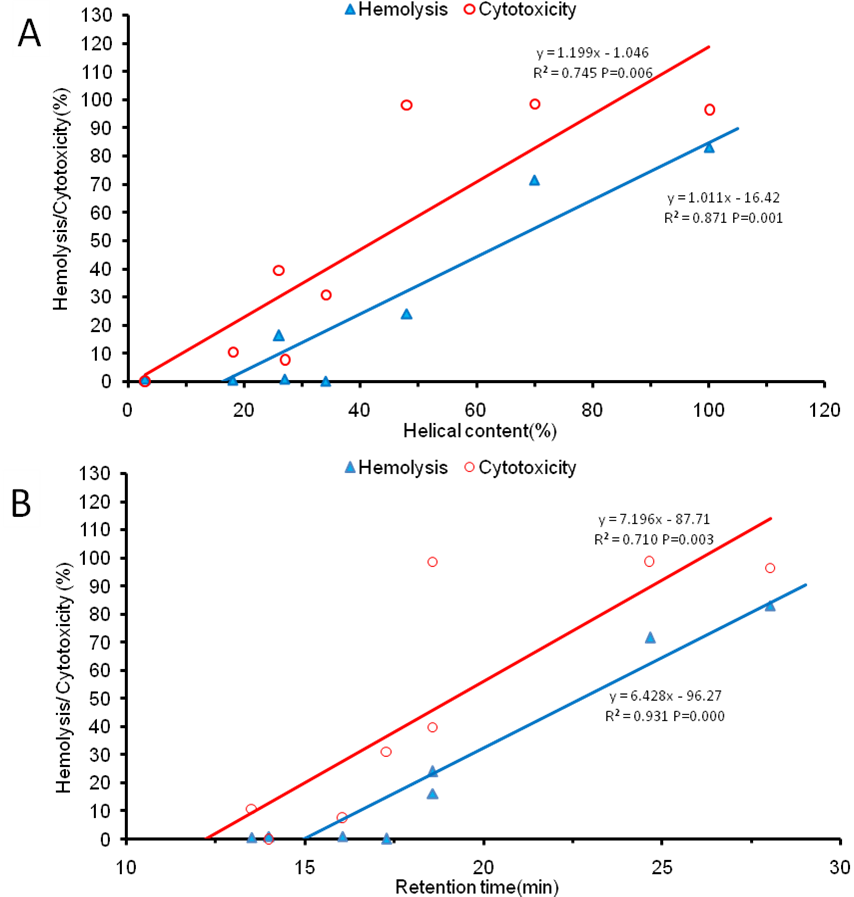


Supplementary Fig. S5. Correlation between the hemolysis / cytotoxicity of the centrosymmetric peptides at 128μM and H values of the centrosymmetric peptides (A) or helical content of the centrosymmetric peptides in 50% TFE (B). H values were reliably reflected by different HPLC retention times.


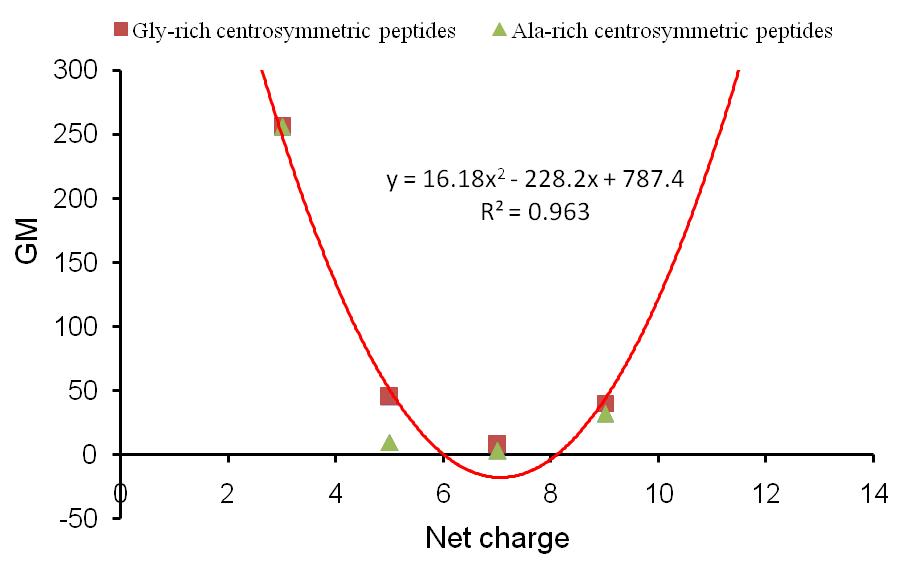


Supplementary Fig. S6. Correlation between the net charge and GM values of the centrosymmetric peptides. GM means the geometric mean of the MBCs of all bacterial strains observed.
